# Psychosocial and environmental risk factors of obesity and hypertension in children and adolescents—a literature overview

**DOI:** 10.3389/fcvm.2023.1268364

**Published:** 2023-11-20

**Authors:** Małgorzata Wójcik, Julio Alvarez-Pitti, Agnieszka Kozioł-Kozakowska, Michał Brzeziński, Rosita Gabbianelli, Vesna Herceg-Čavrak, Elke Wühl, Ignacio Lucas, Dragan Radovanović, Anette Melk, Beatriz González Lopez-Valcarcel, Fernando Fernández-Aranda, Artur Mazur, Empar Lurbe, Claudio Borghi, Dorota Drożdż

**Affiliations:** ^1^ Department of Pediatric and Adolescent Endocrinology, Chair of Pediatrics, Pediatric Institute, Jagiellonian University Medical College in Kraków, Kraków, Poland; ^2^ Interclinical Center for the Treatment of Childhood Obesity, University Children’s Hospital of Kraków, Kraków, Poland; ^3^ Pediatric Department, Consorcio Hospital General, University of Valencia, Valencia, Spain; ^4^ CIBER Fisiopatología Obesidad y Nutrición (CIBEROBN), Instituto de Salud Carlos III, Madrid, Spain; ^5^ INCLIVA Biomedical Research Institute, Hospital Clínico, University of Valencia, Valencia, Spain; ^6^ Department of Pediatrics, Gastroenterology and Nutrition, Pediatric Institute, Jagiellonian University Medical College in Kraków, Kraków, Poland; ^7^ Department of Pediatrics, Gastroenterology, Allergology and Pediatric Nutrition, Medical University of Gdansk, Kraków, Poland; ^8^ Unit of Molecular Biology and Nutrigenomics, School of Pharmacy, University of Camerino, Camerino, Italy; ^9^ Faculty of Health Science, Libertas International University, Zagreb, Croatia; ^10^ Division of Pediatric Nephrology, Center for Pediatrics and Adolescent Medicine, Heidelberg University, Heidelberg, Germany; ^11^ Department of Clinical Psychology, University Hospital of Bellvitge-IDIBELL, Barcelona, Spain; ^12^ Clinical Psychology Unit, University Hospital of Bellvitge, Barcelona, Spain; ^13^ Psychoneurobiology of Eating and Addictive Behaviours Group, Bellvitge Biomedical Research Institute (IDIBELL), Barcelona, Spain; ^14^ Department of Medical Sciences, Faculty of Sport and Physical Education, University of Niš, Niš, Serbia; ^15^ Children’s Hospital, Hannover Medical School, Hannover, Germany; ^16^ Department of Quantitative Methods for Economics and Management, University of Las Palmas de Gran Canaria, Las Palmas de Gran Canaria, Spain; ^17^ Department of Clinical Sciences, School of Medicine and Health Sciences, University of Barcelona, Barcelona, Spain; ^18^ Department of Pediatrics, Pediatric Endocrinology and Diabetes, Medical Faculty, University of Rzeszów, Rzeszów, Poland; ^19^ Department of Medical and Surgical Sciences, University of Bologna, Bologna, Italy; ^20^ Department of Pediatric Nephrology and Hypertension, Chair of Pediatrics, Pediatric Institute, Jagiellonian University Medical College, Kraków, Poland

**Keywords:** children, obesity, hypertension, psychosocial factors, environmental factors

## Abstract

Childhood obesity has become a worldwide epidemic in the 21st century. Its treatment is challenging and often ineffective, among others due to complex, often not obvious causes. Awareness of the existence and meaning of psychosocial and environmental risk factors seems to be an essential element in the prevention and treatment of obesity and its complications, especially arterial hypertension. In this review, we will discuss the role of that risk factors linking obesity and increased cardiovascular disorders including the role of nutritional factors (including the role of unhealthy diet, inadequate hydration), unhealthy behaviors (e.g. smoking, alcohol and drugs, sedentary behavior, low physical activity, disrupted circadian rhythms, sleep disorders, screen exposure), unfavorable social factors (such as dysfunctional family, bullying, chronic stress, mood disorders, depression, urbanization, noise, and environmental pollution), and finally differences in cardiovascular risk in girls and boys.

## Graphical Abstract

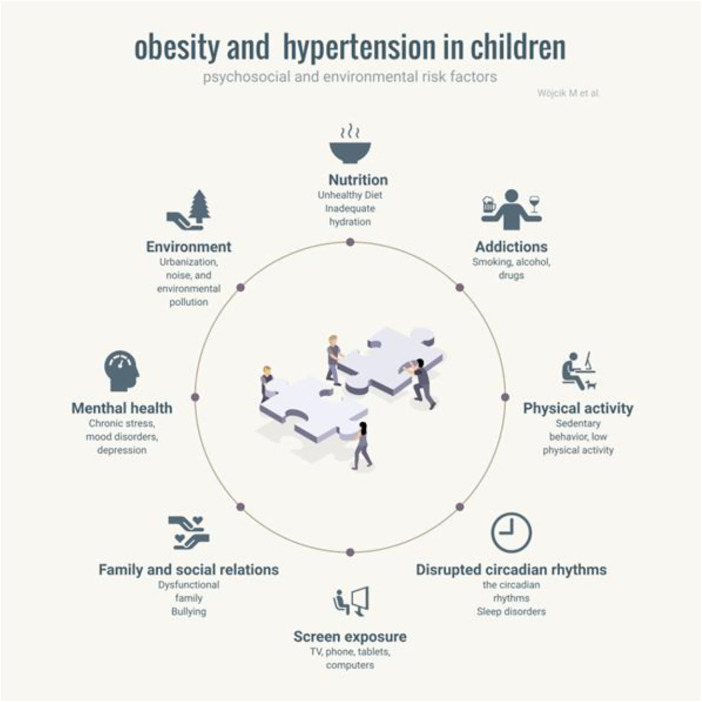

## 1. Introduction

Childhood obesity has become one of the most serious global health crises of this century. According to the most actual definition, obesity is chronic, progressive, relapsing, and treatable multi-factorial, neurobehavioral disease, wherein an increase in body fat promotes adipose tissue dysfunction and abnormal fat mass physical forces, resulting in adverse metabolic, biomechanical, and psychosocial health consequences (1, 2). It is one of the most important reasons for increasing cardiovascular risk in young adults nowadays. Obesity-related hypertension is the dominant form of abnormal, elevated blood pressure (BP) in adolescents. Numerous studies have shown the existence of a tracking phenomenon, in which pathological processes begin as a result of various risk factors and lead to permanent changes and cardiovascular complications through endothelial damage, vascular and myocardial remodeling, and atherosclerotic processes (3). Therefore, targeting obesity reduction solely at children with obesity or overweight may not substantially reduce the overall burden of obesity-related disease in adulthood. Obesity should be considered a multifactorial disease, resulting from a dynamic interplay between genetics, physiology, behavior, psychosocial factors and environmental factors. All these factors accumulate over time since conception and can lead to the deterioration in energy homeostasis throughout life. Most of the reviews published so far focus on factors directly related to the person suffering from obesity: genes, metabolic disorders, and mental factors. The purpose of this study is to provide an up-to-date review of research on external factors—related to the patient's environment. Increased evidence points to their importance. The paper presents a narrative review of the literature on these issues.

## 2. Nutrition related factors

### 2.1. Unhealthy diet

An unhealthy diet in children and adolescents has been associated with overweight and obesity. A major contributor to obesity and hypertension is the high consumption of processed and fast foods high in sodium, saturated- and trans-fats, sugars and calories. Also, beverage like soda, fruit juices, and sports drinks stand for a high font of simple carbohydrates. Song et al., show in their meta-analysis that the prevalence of childhood hypertension correlates with higher body mass index (BMI) (4). In children aged 6–12 years triglyceride levels (≥150 mg/dl), and low-density lipoprotein cholesterol level (≥110 mg/dl) have been positively associated with hypertension, while dietary intake of fiber, vitamin C and copper were negatively related to hypertension (5). Meta-analysis shows a positive association for the risk of hypertension for 100 g/day of red meat or 50 g/day of processed meat (6). Preventive strategies to contrast hypertension include the correct consumption of vegetables and fruits which contain important bioactive molecules, vitamins and fiber useful to modulate properly gut microbiome and help to maintain lower BP. An adequate intake of whole grains is another source of insoluble fibre that can help reduce the risk of developing hypertension (7). A daily consume of 30 g/day of fiber in patients with hypertension (stage 1; 18–65 years old) lower heart rate, contributing to the control of hypertension (8). A meta-analysis on the impact of dietary fiber consumption on hypertension, shows that fiber decreases BP, low-density lipoprotein, plasma cholesterol level and improves blood glucose level (9). The intake of various types of vegetables (3 portions/day) and the correct quantity of fruit (2 portions/day) is associated with short chain fatty acids (SCFA) production from fiber by gut microbiome; SCFA can mediate anti-inflammatory response, regulate lipid and carbohydrate metabolism. Moreover, the bioactive compounds contained in plant-based food can modulate properly genes driving antioxidant, anti-inflammatory and detoxifying responses. Furthermore, green vegetables are key font of folic acid, that together with B2, B6 and B12 (from animal food), choline and betaine, guarantee the substrates and co-factors required by one-carbon cycle, which produces the universal methyl group donor the S-adenosyl-methionine. This pathway permits to convert homocysteine to methionine and guarantees the required methyl groups for the maintenance of cell epigenetic signature. Low-fat dairy products, protein from plant-based food (i.e., legumes and cereals) or from selected animal products (i.e., small fish and white meat) can contribute to maintain a healthy lipid profile. Moreover a positive impact on kidney function (8% lower risk) was measured in individuals consuming omega-3 polyunsaturated fatty acids from seafoods (10). In conclusion the correct lifestyle (food intake and physical activity) can prevent obesity and hypertension in children and adolescents; education needs to start from both parents and children. It is necessary to address the required evidence about healthy (i.e., whole cereals, vegetables, fruit, fish, water) and unhealthy (i.e., processed food, red and processed meat, beverages) food and beverage to stakeholders in order to turn prevention into a strategy for combating obesity and high BP in children and young people.

### 2.2. Inadequate hydration

Even small chronic dehydration can have an impact on both physical health (kidney stones, constipation, urinary tract infections) and mental health. This condition increases fatigue and reduces mental performance by reducing memory, attention, and response time. According to studies, children drink too little water, contrary to the EFSA recommendations (see Table 1).

**Table 1:** Review of studies on hydration status and fluid intake in children.

References	Methodology	Results
Bonnet et al. (11)	Breakfast diary	As in third of the examined children had high urine osmolality (801 and 1,000 mmol kg-1), and total water intake (TWI) was inversely correlated with high urine osmolality.
Bougatsas et al. (12)	Fluid intake from two days	Drinking water and milk correlated with better hydration status, while drinking sweetened beverages was associated with lower levels of hydration (*p* = 0.001)
Kavouras et al. (13)	Two days’ fluid intake	Insufficient water intake was associated with lower hydration levels in children
Kozioł-Kozakowska et al. (14)	School day urine osmolality	53% of schoolchildren were insufficiently hydrated and 16.3% of them were very dehydrated (urine osmolality > 1,000 mOsm/kgH2O)
Michels et al. (15)	Food frequency questionnaire FFQ for children	Children were at high risk of dehydration (school urine osmolality 888 mmol kg−1)
Padrão et al. (16)	Twenty-four-hour dietary interview	More than half of the children were under-hydrated; higher water intake was associated with better levels of hydration
Stahl et al. (17)	Three-day nutrition diary of weighed foods	Proper hydrated children had a higher total water intake with diet and a lower energy density of the diet compared to less hydrated children.
Stookey et al. (18)	Breakfast diary	Increased urine osmolality (>800 mmol kg-1) was associated with lower water consumption in the morning

As water turnover increases with the BMI, children with obesity need more water. Studies have shown that children with obesity are more likely to dehydrate (19, 20). Children with obesity drink less liquids, have lower total body water percentages, and have higher urine density, while children with normal weight are less dehydrated (21). Additionally, dehydration may be aggravated due to the increased sodium intake in children with obesity. Studies in the pediatric population show an increase in sodium intake with an increase in BMI *Z*-score. Sodium intake, based on 24-h urinary excretion, has been associated with obesity in several European studies, with those with the highest sodium excretion having a higher BMI compared to those with the lowest excretion. A Polish study found that high sodium levels in overweight children and excessive salt consumption, as well as low potassium consumption, are significant predictors of dehydration regardless of total water consumption (TWI) (22). In this study, the systolic BP was also directly associated with 24-h urinary sodium excretion. Therefore, nutritional education of children with obesity should focus on increased consumption of products rich in potassium, mainly vegetables and fruits, in order to compensate for the effect of increased sodium intake. Children should be also educated on how much and what liquids they should choose to drink.

### 2.3. Smoking, alcohol and drugs

According to the Organization for Economic Co-operation and Development (OECD) report on adolescent smoking and alcohol consumption published in 2021, the average prevalence of smoking among adolescents in all OECD countries was 16.4%. On average, 21.5% of adolescents aged 15 years had been drunk at least twice in their lifetime, with prevalence only slightly higher among boys (22.6%) than girls (20.3%), and 7% of 15-year-olds reported cannabis smoking within the last months (23). The adverse effects of tobacco smoking on adult cardiovascular health are well described. However, these negative effects are not limited to nicotine use in adolescence and adulthood, but also to fetal nicotine exposure during pregnancy or passive smoking in infancy and childhood (24–28). In a meta-analysis of 17 studies on the effects of maternal smoking during pregnancy, children with intrauterine nicotine exposure had an increased risk of obesity at an average age of nine years compared with children of nonsmoking mothers (29). In another meta-analysis, based on results of more than 84,000 children reported in 14 observational studies, children whose mothers smoked during pregnancy were at 50% elevated risk for overweight, compared with children whose mothers did not smoke (30). It is suggested that maternal smoking during pregnancy may lead to nicotine-induced fetal growth retardation and faster postnatal weight gain, both of which are associated with an increased risk of obesity later in life (30). In addition, maternal tobacco smoking may increase the child's BP by inducing endothelial and vascular dysfunction and alterations of renal morphology and function (31). Maternal smoking has also been shown to be associated with reprogramming of BP control mechanisms in the child (32). Both active and passive smoking have been associated with higher systolic BP levels in children and adolescents (26). Smoking exposure even at low levels and intensity of alcohol use were associated individually and together with increased arterial stiffness (33). Starting to smoke in adolescence is associated with an increased risk of nicotine dependence, both in childhood and thereafter. And although e-cigarettes seem to be a less harmful form of nicotine delivery, they appear to increase the risk of nicotine dependency later in life (34). Recently, it has also been shown that smoking and alcohol consumption are independent risk factors influencing childhood obesity (35). Beliefs that smoking protects against obesity may be over-simplistic; especially among younger and heavier smokers. Among smokers, the risk of obesity increased with the amount smoked and former heavy smokers were more likely to be obese than former light smokers (adjusted OR 1.60) (36). In a meta-analysis of 21 studies, active smoking increased the risk of childhood obesity by 17%. In a bidirectional analysis of the correlation between BMI and smoking, a higher BMI did not predict an increased risk for cigarette smoking, while cigarette smoking predicted a higher BMI, and higher frequency of cigarette smoking in adolescence predicted chronic obesity in young adulthood (37–39). Given the demonstrated correlation between e-cigarette use and cigarette smoking among adolescents, the association between obesity and cigarette smoking could also apply to e-cigarette use (40). In contrast to the effect of smoking on BMI, based on a meta-analysis of 13 studies, drinking alcohol had no significant effect on the risk of childhood obesity (35). However, alcohol energy (7 kcal/g) can be a contributing factor to weight gain. Therefore, alcohol consumption might increase the risk of obesity in some individuals. Smoking promotes damage to the cardiovascular system in several ways. Tobacco smoke is a complex mixture of more than 5,000 substances, including numerous carcinogenic and toxic substances. These can interact with each other and reinforce effects. Nicotine constricts blood vessels by releasing catecholamines, thereby acutely increasing BP. Several different constituents of tobacco smoke damage the endothelium, induce dyslipidemia, have prothrombotic effects, and cause chronic inflammation. Active smokers have increased arterial stiffness compared with nonsmokers, promoting chronic hypertension. The deleterious association between tobacco exposure and endothelial function is observed even in young children without other risk factors (41). The tobacco consumption during adolescence is the greatest risk factor for cardiovascular disorders and events in adulthood (42).

Acute alcohol consumption results in a short-lasting decrease in BP (43). In contrast, chronic alcohol consumption has a dose-dependent, linear association with hypertension that has been shown to be consistent across sex, age and ethnicity, and is independent of BMI, smoking habits, and physical activity level (44–47).

Smoked cannabis is one of the most commonly used drugs among young people. Acute cannabis smoking has been associated with an increased risk of heart attacks and ischemic strokes. However, it is still uncertain whether individuals who smoke cannabis exclusively are at increased risk for accelerated atherosclerosis, as has been found in tobacco smokers. The stimulants methamphetamines and cocaine increase the risk of stroke and heart attack and accelerate atherosclerosis (48).

## 3. Sedentary behavior, low physical activity

### 3.1. Definitions

A wealth of evidence supports the importance for children and adolescents to meet physical activity guidelines, which include 60 min of moderate to vigorous physical activity (MVPA) daily (49). However, research on the associations between physical activity (PA) and health for the potentially remaining 23 h per day has traditionally been less numerous but is growing rapidly. Sedentary behavior is particularly important, defined as any waking behavior characterized by energy expenditure ≤1.5 MET while sitting or reclining, as defined by the Sedentary Behavior Research Network (SBRN) in 2012, in an effort to have a consensus definition (50). However, there are some terms such as physical inactivity, screen time, stationary behavior etc. that can lead to confusion. They have been cleared on a recent document published by the same network in 2017 (51). Definitions are included in Table 2. Another point of discussion was if the general definition of sedentary behavior could also be applied to children, considering their higher basal metabolic rate. There was broad support for using also in preschool children and youth the threshold of ≤1.5 METs to define sedentary lifestyle provided that a standard higher than 3.5 ml/kg/min is used to define MET, following the recommendations of the compendium of energy expenditures for youth (52–54).

**Table 2:** Final definitions of key terms from the sedentary behavior research network (SBRN) terminology consensus project (51).

Term	Definition
Sedentary behavior	Sedentary behavior is any waking behavior characterized by an energy expenditure ≤1.5 metabolic equivalents (METs), while in a sitting, reclining or lying posture. - **Sedentary time (ST):** The time spent for any duration (e.g., minutes per day) or in any context (e.g., at school or work) in sedentary behaviors. - **Sedentary bout:** A period of uninterrupted sedentary time. - **Sedentary interruptions/breaks:** A non-sedentary bout in between two sedentary
Physical inactivity (PI)	An insufficient physical activity level to meet present physical activity recommendations. General definition applies to all age and ability groups.
Stationary behavior	Stationary behavior refers to any waking behavior done while lying, reclining, sitting, or standing, with no ambulation, irrespective of energy expenditure.
Sedentary behavior pattern	The manner in which sedentary behavior is accumulated throughout the day or week while awake (e.g., the timing, duration and frequency of sedentary bouts and breaks). **Prolonger:** Someone who accumulates sedentary time in extended continuous bouts. **Breaker:** Someone who accumulates ST with frequent interruptions and in short bouts.
Standing	A position in which one has or is maintaining an upright position while supported by one's feet.
Screen time (ST)	Screen time refers to the time spent on screen-based behaviors. These behaviors can be performed while being sedentary or physically active.
Sitting	A position in which one's weight is supported by one's buttocks rather than one's feet, and in which one's back is upright.
Reclining	Reclining is a body position between sitting and lying.
Lying	Lying refers to being in a horizontal position on a supporting surface.

MET, metabolic equivalent corresponding to resting metabolic rate of the population under study.

### 3.2. Epidemiology of sedentary behavior in children and adolescents

Focusing on insufficient PA, in 2010 worldwide, its prevalence in children aged 11–17 years was 78.4% for boys and 84.4% for girls (55). The same research group reported global trends of non-objectively measured PA from 2001 to 2016, based on 298 surveys from 146 countries, including 1.6 million participants (56). In 2016, 81% of the participants between 11 and 17 years old, presented insufficient PA. Between 2001 and 2016, the prevalence decreased by 2.5% (significant change) for boys, while there was no significant change for girls. This makes the difference in PI between sexes increase to 7.1% (4.7% in low-middle-income countries and 11.8% in high-income countries). No differences were found in PA according to the income of the countries in which they live. The gold standard measurement to determine sedentary time (ST) is accelerometry. The International Children's Accelerometry Database collects standardized PA data measured by the actigraph accelerometer. In 2020 this group published the pooled results of eight studies including longitudinal accelerometer data of 5,991 children (aged 4–15 years), with a mean follow-up of 2.7 years (57). The authors found that the average total ST (from 7am to midnight) of the participants ranged from 243 to 375 min/day at baseline for boys and from 250 to 393 min/day for girls, with significant but small in size differences among sexes. In both sexes, the average ST far exceeds the maximum of 120 min recommended by the WHO. The results of the multilevel analyses indicated that total ST increased on average by 21.4 min/day for each additional year of follow-up, being this increase high during childhood and moderate during the transition from childhood to adolescence. And, are there differences in ST between normal weight and children and adolescents with obesity? This question was answered by this systematic review including 14,739 PA-accelerometer-measured participants (3,526 with obesity) (58). Researchers did not find marked differences in ST between obese and non-obese peers, although the amount of moderate-to-vigorous intensity physical activity (MVPA) was superior in the most ST and also by screen time of the included studies in normal-weight participants. Contrary to what common sense would lead us to believe, the evidence supports that unfortunately, among children and adolescents, sedentary behavior is the norm, not the exception. Overall, emerging evidence in the wake of the COVID-19 pandemic indicates that the number of children and adolescents meeting guidelines for physical activity and sedentary behavior is declining (59, 60). The COVID-19 pandemic has led to the implementation of policies that mandate various restrictions on daily life, including social distancing, the closure of public services and schools, and movement limitations. The coexistence of childhood obesity and COVID-19 and changes in the bioecological environment have put children and adolescents at increased risk for developing obesity and exacerbating the severity of this disorder (61). The need for social isolation had an effect of causing or worsening obesity and its comorbidities (62).

### 3.3. Effect of sedentary behaviour on health

In children and adolescents sedentary behavior is linked to an increased risk to develop obesity, increased cardiovascular risk, decreased fitness, behavioral problems, and lower self-esteem (63). Among the mechanisms proposed to explain these associations, are the decreased energy expenditure caused by SED and also by screen time in relation to the increase in the consumption of unhealthy foods (64). Evidence about the direct unfavorable effects on metabolic health is scarce and partly it seems to be mediated mainly by screen-time-unhealthy-eating (65). In both cross-sectional and longitudinal studies, a relationship has been found between excessive inactivity time and higher risk to develop HTN. Recent studies in adults based on objective measures of physical activity indicate that sedentary behavior is associated with an increased risk of obesity, CVD, decreased CRF and all causes of mortality even in subjects who comply with physical activity recommendations. Only, high levels of moderate-intensity physical activity (i.e., about 60–75 min per day) seem to eliminate the increased risk of death associated with high sitting time (66). Further analysis of the data indicates that this is due in part to the negative effect of prolonged uninterrupted ST time bouts (low number and duration of breaks in ST time). Recent studies in adults have shown that these prolonged periods have deleterious effects on health, independent of those produced by physical inactivity (67) because in addition, they affect vascular function, altering systemic blood flow (68) they favor an increase in BP they have been related to the activation of the sympathetic system (69) they raise postprandial glycemia and insulin resistance by reducing consumption by inactive muscles (70) they alter the regulation of cerebral blood flow (71) they favor an increase in systemic inflammation (67, 72) and, finally, they could even reduce the beneficial effect of the practice of physical activity (73). Among ST behaviors in children and adolescents, recreational screen time (63, 74) and prolonged uninterrupted ST bouts (75) have shown associations with adverse health outcomes. This association was directly correlated to the duration of ST bouts and indirectly with the mean duration of sedentary breaks. In the case of young children, this negative effect of prolonged periods of inactivity has not been demonstrated fully, because the natural behavior of children is to spontaneously interrupt periods of inactivity. In children (5–12 years old) total MVPA is important for lowering cardiometabolic risk in children, while both total and uninterrupted sedentary time seem of less importance (76, 77). But the social trend of continuous use of screens or prolonged periods of sitting during classes could also have an impact on child´s health. At school, with age, the number of hours spent at school and the number of hours needed for homework and self-study also increases. For this reason, the amount of forced time spent sitting increases during adolescence. In addition, these prolonged periods of inactivity have been identified in higher-risk populations such as children and adolescents with obesity or adolescents with intensive screen use (more on weekends) but more research is needed on this topic.

Most of these deleterious effects can be avoided by interrupting these prolonged periods of inactivity. Therefore, recommendations are increasingly being made not only to increase PA by decreasing ST and reaching the PA goals but also to interrupt prolonged periods of inactivity with small PA “snacks” (78, 79). Strategies such as “sitting less and moving more” or “physical activity snacks” (79) could be adapted to pediatric populations pursuing a goal: replacing sedentary behavior with any intensity of physical activity (that is, movement) that will have health benefits, but with greater benefits seen when sedentary behavior is replaced with moderate-vigorous- intensity physical activity (80, 81). This type of intervention would be even more important in the case of children and adolescents with obesity for whom introducing and maintaining MVPA is complicated. A school and/or family-level intervention focused on reducing ST (and prolonged periods) through short periods of intense PA could be a successful alternative. The effect of this type of intervention to reduce sedentary behaviour on blood pressure levels in children and adolescents has not been rigorously evaluated. We can only say that there is good evidence that PA of at least moderate to vigorous intensity has a beneficial effect on blood pressure control in youth with hypertension.

### 3.4. Screen exposure

Screen time has become a ubiquitous part of modern childhood. Many children are spending hours each day in front of screens watching TV, using a smartphone, computer or playing video games. The American Academy of Pediatrics and the World Health Organization (WHO) have recommended avoiding screen use completely in children under 18 to 24 months and limiting screen time to 1 h a day in children aged 2–5 years (49). While there is no agreement on the amount of leisure screen time that is considered to be harmful for older children, it is generally suggested that this time should be limited to two hours per day. Multiple studies have shown a positive association between screen time and both hypertension and obesity in children (82, 83). The connection between increased screen time and obesity and higher BP levels could be due to various factors. Excessive screen time leads to less physical activity, and very often to eating large amounts of food, usually unhealthy snacks and sugary drinks which can promote weight gain (84). The marketing of unhealthy foods and drinks through screen-based media may also be contributing to the development of obesity. Furthermore, screen time can lead to disrupted sleep patterns, which can have negative effects on both hypertension and obesity (82). In addition, the habit of excessive TV viewing in younger children is tracking to later childhood and adolescence and thus exposes the child to a long period of unhealthy, sedentary behavior (85). Prolonged TV viewing and total screen time during leisure time in adolescence are associated with unfavorable levels of several cardiovascular risk factors in young adulthood. Nagata et al. conducted a study with the aim of determining the potential links between screen time in childhood and cardiometabolic risk in adulthood over a 24-year period. Their findings indicated that greater screen time during adolescence was associated with an increased likelihood of certain cardiometabolic disease indicators in adulthood (86). Hagjoo et al. in a meta-analysis of a large sample size (*n *= 112,489) also reported that high screen time is associated with a 1.27 times higher chance of overweight/obesity in adolescents, with TV watching being the most influential factor (87). Zulfiqar et al. reported a positive association between obesity and TV watching, but not with video games (88). According to Lopez Gonzalez's research, in a population-based cross-sectional study that evaluated 1,463 children and adolescents, high screen time was identified as obesogenic (89). Stabouli et al. found that screen time is linked to hypertension and obesity in children. Each additional hour of weekly screen time increased the likelihood of hypertension by 1.18 times in adolescents, even after adjusting for other risk factors (90).

## 4. Disrupted circadian rhythms

### 4.1. The circadian rhythms

The circadian rhythms are driven by a central clock located in suprachiasmatic nucleus and various peripheral clocks located in different tissues and organs of the body (91). The process of metabolism is also under circadian regulation. Disturbances of synchronization between the internal clock and environmental timers result in disruption of the circadian rhythms that seriously impact metabolic homeostasis leading to changed eating behavior, altered glucose and lipid metabolism, and weight gain. This in turn augments the risk of having various cardio-metabolic disorders such as obesity, diabetes, metabolic syndrome, and cardiovascular disease. Blood pressure follows a circadian rhythm, it increases on waking in the morning and decreases during sleeping at night (92). Disruption of the circadian BP rhythm has been reported to be associated with worsened cardiovascular and renal outcomes. Growing evidence from animal models has found that the circadian rhythms of BP can be distinctly altered by genetic manipulation of the core clock genes (93). An explicit mechanism has not been explained between chronic kidney disease and disruption of circadian BP rhythm. It has been shown, that an excessive circadian variation in BP is also associated with an increased risk of nephropathy. Probably due to this data, the application of anti-hypertensive drugs according to this information should be change for rational and effective administration time.

### 4.2. Sleep disorders

The most overt circadian rhythm is the sleep–wake cycle. Healthy night sleep is necessary for the full functioning of the brain, memory, regulation of metabolic processes, maturation and growth. Sufficient sleep is essential for maintaining healthy physical, mental, and emotional functioning. It has been shown by The National Health and Nutrition Examination Survey (NHANES) that there is a link between too few hours of daily sleep at night and a higher prevalence of obesity (94). The relationship between sleep disorders and increased cardiovascular risk in adults is clear. The literature on cardiovascular consequences of sleep disorders in children is not as robust, but there is some evidence of BP disturbances in children with sleep deprivation and obstructive sleep apnea (95). An inadequate number of hours of sleep or its disturbed course cause adverse effects on mood and behavior control, overexcitement in the cognitive-emotional and behavioral spheres, as well as in the autonomic or central nervous systems, and hyperactivation of hypotalamic-pituitary-adrenal axis (96). This is manifested in an increase in heart rate and a decrease in heart rate variability during the day, an increase in body temperature, and an increase in the ACTH and cortisol secretion (97). The same mechanisms contribute to the development of arterial hypertension. The prevalence of arterial hypertension in children with sleep-disordered breathing is 14%, which is almost three times more than in the general population (98). On the other side, it has been demonstrated by Hinkle et al, that 23% of children with elevated BP snored, and in those that underwent polysomnography, 80% were diagnosed with obstructive sleep apnea (99). In another study, it was shown that children with apnea/hypopnea index (AHI) > 5 had higher prevalence of systolic hypertension (57%) when compared with non-snorers or children with AHI < 5 (100). Children who sleep less than the age-adjusted amount of sleep (9–11 h for children at the age of 6–12, 8–10 h for older adolescents) are also more likely to have high BP (101). As it has been revealed less than a third of adolescents in the USA obtain the recommended amount of sleep for age on school nights (102). During adolescence the circadian timing system undergoes a phase delay. For this reason, the time to fall asleep in the evening and the time to wake up naturally in the morning are naturally delayed. This, together with the need to adapt to the rhythm imposed by social functioning and school, can reduce the number of hours of sleep. In addition, in the evening and night using the screens, social-network engagement become more available as youngsters pass through adolescence (103).

## 5. Family and social relations

### 5.1. Unfavorable family interactions

Frequent and unfavorable interactions within family units are associated with a higher risk for chronic illnesses, such as cardiovascular disease (104). The family environment and negative childhood events significantly influence the development of cardiovascular disease (105). Dysfunctional family and negative family dynamics play a significant role in the development of hypertension (106). Previous 23-year longitudinal research found that negative experiences predict high BP (107). Additionally, lower BP in children aged 15 was predicted by parental sensitivity (i.e., observer assessments of emotional support during a difficult task) in the early years (108). Moreover, family connection dynamics and poor sleep quality both raise the risk of hypertension (109).

### 5.2. Bullying

Bullying at school between classmates is a globally acknowledged problem (110). Bullying is understood as repeated physical or verbal abuse where the aggressor is more dominant than the victim (111). Obesity in children and adolescents is linked to worse quality of life and more stigma or unfair treatment. Decreased school motivation, sadness, depression, psychosomatic issues, loss of sleep, and even suicide are possible outcomes for bullied individuals (112). A meta-analysis indicates that both youths with obesity or overweight experience significantly more bullying than normal-weight youths (113). To our knowledge, no research has directly identified bullying at school as a risk factor for hypertension in children and adolescents. However, several studies have found a link between experiencing bullying and reporting poor general health and also with exhibiting unhealthy dietary habits (114–123).

### 5.3. Chronic stress, mood disorders, depression

The American Heart Association published a scientific statement in 2015 that recognized mood disorders, such as major depressive disorder or bipolar disorder, as moderate-risk conditions for early cardiovascular disease among youth (124). Since then, several cross sectional and longitudinal studies endorsed this statement, reporting evidence of the association between mood dysregulation and medical conditions, such as obesity or hypertension, in children and adolescents. A study conducted in Turkey, the European country with the highest rates of overweight and obesity, found increased eating behaviours associated with negative feelings in preadolescents, possibly as a coping strategy, that can result in weight gain (125, 126). Regarding the direct relationship of mood disorders with obesity and hypertension in children and adolescents, Medrano et al. reported results about cardiovascular risk factors in youth with overweight and obesity (127). In this population, they found that mood disorders were associated with elevated adiposity levels and higher rates of hypertension, although those with mood disorders were also older. Moreover, another cross-sectional study found that adolescents and young adults with bipolar disorder showed twice the prevalence of metabolic syndrome than the general population (128). Also, in relation to cardiovascular diseases (CVD), Waloszek et al. found that adolescents with depressive symptoms had poorer vascular functioning and higher risk of CVD than adolescents without depressive symptoms, and Korczak et al. reported that children and adolescents with depression presented a higher rates of early CVD among family members (129, 130). Longitudinal evidence has also been reported in this regard. A prospective cohort study with adolescents found a relationship between high levels of depression, obesity and poor dietary and physical activity behaviours, reinforcing the idea of a negative feedback loop between depressive symptoms, poor diet, low physical activity and obesity (131). However, another prospective study reported that female children with higher negative affect showed decreased physical activity in the longitudinal follow-up, but male children showed the inverse effect, with increased physical activity associated with higher negative affect suggesting sex differences in the relationship of these factors. Chronic emotional stress is another relevant factor that has also been associated with the cardiovascular health of young people as it may result into the development of obesity and/or hypertension suggesting sex differences about the relationship of these factors (132–136). A longitudinal study reported that adverse experiences, that can result in chronic stress, were associated with the development of obesity during childhood, even when these events occurred at very early stages of the development (137). In the same line, another longitudinal study also assessed the relationship between child adverse events and BMI and found a relationship between adverse events during childhood or adolescence and increased adult BMI (138). This would suggest that the negative impact of the stress response to adverse events might take some time to express in the BMI. However, other recent prospective results suggest that obesity may be driving stress related biological factors, rather than preceding them (139). Socioeconomic adversities could also produce elevated chronic stress during childhood and adolescence and, therefore, be a risk factor to develop obesity during childhood (134). However, Olstad et al. found no relation between hair cortisol levels and BMI in children with low socioeconomic status, suggesting that obesity development would be more related to psych behavioural factors (140). However, another study found that racial discrimination and low household education were associated relationship with BMI in children and adolescents, but not the perceived stress (141). Both mood and chronic stress seem to have an impact on cardiovascular health of children and adolescents. They may produce a direct effect by disturbing the physiological and psychological functioning, and also an effect via unhealthy behavior such as poor sleep patterns, low physical activity, unhealthy eating or consumption of alcohol and tobacco (142).

### 5.5. Social environment

The social environment is also extremely important in the prevention, treatment of obesity and the prevention of its complications. Appropriate social conditions and support for children from risk groups are of great importance. Building public–private partnerships, engaging all entities to cooperate, in particular nongovernmental organizations promoting a healthy lifestyle, consumers, organizations, and private sector entities, including the food industry and media—joint activities to promote healthy behavior (143).

## 6. Urbanization, noise, and environmental pollution

Changes related to the development of civilization may be important factors in increasing the frequency of non-communicable diseases in pediatric population, including arterial hypertension. Emerging risk factors include pollution (air, water, noise, and light), urbanization, and a loss of green space. This effect may be direct or indirect due to higher prevalence of obesity and overweight. Studies of recent years, mainly from Asian or African countries, clearly show that the progressing urbanization process, sedentary lifestyle, and poor dietary habits are a few new factors that might contribute to a higher prevalence of these problems not only in adults, but also in children (144). Recent findings demonstrate also that such endocrine-disrupting chemicals, termed “obesogens”, can promote adipogenesis and cause weight gain. This includes compounds to which the human population is exposed in daily life through their use in pesticides/herbicides, industrial and household products, plastics, detergents, flame retardants and as ingredients in personal care products (145). A study conducted in Ghana found that BP levels were lower in the rural population than in the semi-urban and urban children (146). Research conducted in China confirmed that in comparison with urban children, rural children had a lower prevalence of arterial hypertension (especially isolated diastolic hypertension) (147). Finally, as shown in a recently published meta-analysis by Meena et al. arterial hypertension in India is diagnosed in 7% of urban children 7% (95% CI: 6%–9%), compared to 5% in rural counterparts 5% (95% CI: 3%–6%) (144). Urbanization is undoubtedly associated with increased exposure to noise, mainly from transport, and air pollution. The existing literature of both mechanistic and epidemiological design strongly points towards a relationship between exposure to transportation noise and elevated BP in adults (148). Nevertheless, the association between transportation noise and BP in children has also been investigated. In a meta-analysis by Dzhambov et al. authors reported that a 5 dB rise in road traffic noise at kindergarten/school was associated with a 0.48 mmHg higher systolic BP [95% CI (−0.87–1.83)] and a 0.22 mmHg higher diastolic BP [95% CI (−0.64–1.07)] (149). Many epidemiological studies indicate that nocturnal noise exposure (especially aircraft or railway noise) may be more relevant for cardiovascular health than daytime noise exposure (150). The effect of noise on increasing BP is multidirectional. Starting from sleep, communication and daily activities annoyance. That chronic annoyance causes stress characterized by increased levels of stress hormones such as cortisol and catecholamines, which causes permanent changes leading to an increase in BP (151). Although limited data suggest air pollution exposures may contribute to pediatric high BP, the results of the recently published CANDLE study clearly confirm such a relationship. Moreover, as shown by the authors even prenatal air pollution exposure, particularly in the second trimester, is associated with elevated early childhood BP (152). Short and long-term exposure to air pollution provokes oxidative stress, systemic inflammation, and autonomic nervous system imbalance that subsequently induce endothelial dysfunction and vasoconstriction leading to increased BP (153).

## 7. Differences in cardiovascular risk in girls and boys

Sex differences in BP in children and adolescents are well known (154). In general, girls exhibit lower BP compared to boys. BP rises with age, with this increase not attributed to the process of aging itself, but rather linked to the increase in body size (155). During adolescence the differences in BP between girls and boys become more prominent (156). BP difference between the sexes is primarily influenced by lower stroke volume as well as lower total peripheral resistance. There is a positive correlation between intra-abdominal fat and BP, albeit less significant in girls than in boys. Additionally, factors such as fat-free mass, fat mass, and insulin resistance are also positively associated with BP, with similar patterns observed in both girls and boys (156). These associations, combined with the sex-specific variations in body composition and metabolism, contribute to the observed BP differences between the sexes. In pre-pubertal girls (aged 6–11 years) BP seems to be associated also with age independent of body size and adiposity; this association is not observed in boys (157). Among 6 to 11-year-old children, the prevalence of hypertension is higher in girls compared to boys with a higher prevalence of elevated diastolic BP in girls (158). A retrospective study assessing 74,233 children showed male sex to be associated with high BP and a possible association between material and social deprivation and high BP, particularly in boys (159). A longitudinal study on adolescents (aged 12–18 years) found that boys had a greater likelihood of developing high systolic BP compared to girls (160). These differences regarding BP and hypertension have to be in context with the known differences in obesity. Girls tend to have lower rates of obesity than boys depending on the country (161). Boys typically have more visceral fat, while girls tend to accumulate more subcutaneous fat (162). Despite this, a study showed significant associations of visceral fat with cholesterol subclasses were only in girls (163). Another study reported that girls with higher BMI have a greater left ventricular muscle mass (LVMI) compared to boys with comparable BMI (164). Additionally, in youth, masked hypertension is a precursor of sustained hypertension. The risk of developing sustained hypertension is higher in boys than it is for girls (165). These recent reports challenge the general view that cardiovascular risk is greater in boys, but point towards a greater vulnerability in girls under specific circumstances. More research is needed to address this issue.

## 7. Closing remarks

Obesity is a relevant risk factor whose prevalence is progressively increasing and involves childhood and adolescence with a negative impact for future generations. The problem of obesity in children is that of the associated hypertension and other risk factors for cardiovascular and metabolic disease that have been repeatedly described in cross-sectional and longitudinal epidemiology. The increase in body weight is just the quantifiable evidence of an underlying involvement of cardiometabolic system progressively leading prospectively to an impaired quality of life and general health. This pathophysiological interaction introduces the concept that the management of obesity in childhood should take into consideration a well-designed strategy aimed at controlling body weight but also any additional risk factor involved in cardiovascular and metabolic abnormalities, in particular hypertension. Dietary measures are the cornerstone of prevention through a correct and well-adjusted nutritional approach including healthy foods, proper hydration, and the prevention of dangerous lifestyle habits (smoking, alcohol, drugs). One of the main causes of obesity/hypertension in children is the progressive decrease of physical activity that has been progressively overcome by a remarkable “static” activity based on the extensive use of interacting devices that is clearly expressed by the new measure of physical inactivity that is the duration of “screen time” that can be particularly dangerous when combined with alterations of circadian rhythms. An increase in the exposure to screen-based interfaces particularly during the night or early morning can be responsible for a remarkable disruption of the individual neuro-humoral response that can have a negative impact on energetic metabolism and autonomic response leading to those significant changes in BP organ damage that are frequently observed in overweight adolescent subjects. In particular, the increase in the isolation behaviors either physical (self-restriction) or functional (extensive use of ear sets of any kind) can significantly affect both exercise capacity and mental attitude toward reciprocal negative relationship resulting in a positive energy balance that can promote childhood obesity predisposing to cardio-metabolic disease in adult life. As expected, a cornerstone in the prevention of obesity and its consequences in terms of cardiovascular and metabolic disease is the quality of the “environment” surrounding children and adolescents during their daily life. The most important is the “human” environment that involves both the family and the peers (friends, classmate, teammate, etc). than can directly or indirectly contribute to food disorders but also to the abnormalities of BP and metabolic control with a negative impact on cardiometabolic profile. An improvement in the functionality of family group and a constructive influence by the many daily contacts may largely prevent dangerous behaviors and promote an easy lifestyle with some advantages in terms of weight control As far as the” natural” environment, many recent studies have emphasized the negative impact of air and acoustic pollution on cardiovascular health with a significant increase in the rate of myocardial infarction and stroke in the adult population. Both these risk factors can increase the sympathetic stimulation, the endothelial damage, and the insulin sensitivity with an unfavorable influence on the interaction between environmental risk factors, overweight, and BP. All these specific conditions, combined with the sex-specific variations in body composition and metabolism, may support the observed differences in BP and cardiovascular risk between the sexes. The evidence reported in this paper challenges the general view that cardiovascular risk is greater in boys but point towards a greater vulnerability in girls under specific circumstances and this is something that is needed to be addressed more deeply in the future.

## 8. Conclusion

The problem of obesity and hypertension is progressively growing increasing the risk of cardiovascular disease in the next adult generation. The early identification of the all possible and emerging risk factors in childhood is a cornerstone for present and future approach to cardiovascular prevention in the general population.
